# High mobility group box 1 regulates gastric cancer cell proliferation and migration via RAGE-mTOR/ERK feedback loop

**DOI:** 10.7150/jca.51049

**Published:** 2021-01-01

**Authors:** Tuo Tang, Shengnan Wang, Tianyu Cai, Zhenyu Cheng, Yu Meng, Shimei Qi, Yao Zhang, Zhilin Qi

**Affiliations:** 1 Department of Biochemistry and Molecular Biology.; 2 Anhui Province Key Laboratory of Active Biological Macro-molecules.; 3 School of Clinical Medicine, Wannan Medical College, Wuhu, Anhui 241002, P.R. China.

**Keywords:** High mobility group box 1 (HMGB1), gastric cancer, proliferation, migration, RAGE, Akt/mTOR, ERK

## Abstract

Gastric cancer (GC) is a common malignancy tumour in China. Despite various therapeutic approaches to improve the survival rate of GC patients, the effectiveness of currently available treatments remains unsatisfactory. High mobility group box 1 (HMGB1) is reported to play a role in tumour development. However, the molecular mechanisms involved in HMGB1-mediated regulation of proliferation and migration of GC cells remain unclear. In the present study, we demonstrated that HMGB1 is highly expressed in GC cells and tissue. In HGC-27 GC cells, HMGB1 overexpression or HMGB1 RNA interference both demonstrated that HMGB1 could promote GC cell proliferation and migration. Investigation of the underlying molecular mechanisms revealed that HMGB1 enhanced cyclins expression, induced epithelial-to-mesenchymal transition and matrix metalloproteinase (MMPs) expression and promoted RAGE expression as well as RAGE-mediated activation of Akt/mTOR/P70S6K and ERK/P90RSK/CREB signalling pathways. We also found that inhibition of ERK and mTOR using specific inhibitors reduced recombinant human HMGB1-induced RAGE expression, suggesting that the RAGE-mTOR/ERK positive feedback loop is involved in HMGB1-induced GC cell proliferation and migration. Our study highlights a novel mechanism by which HMGB1 promotes GC cell proliferation and migration via RAGE-mediated Akt-mTOR and ERK-CREB signalling pathways which also involves the RAGE-mTOR/ERK feedback loop. These findings indicate that HMGB1 is a potential therapeutic target for GC.

## Introduction

Gastric cancer (GC) is a common cancer in China, and has high morbidity and mortality rates 1. Due to a lack of early diagnostic markers and easy metastasis, the effects of GC therapy remain unsatisfactory 2. Therefore, it is important to identify a biomarker for effective GC diagnosis and treatment.

High mobility group box 1 (HMGB1) is a highly conserved chromosomal protein located mainly in nuclei and is involved in transcription-level regulation of various genes 3. HMGB1 can be actively or passively released into the extracellular environment, where it binds with its receptors, including receptor of advanced glycation end-product (RAGE) and toll-like receptors and regulates the development of various types of tumour 4, 5. The effect of HMGB1 on malignancy is complicated since it both promotes and counteracts malignancy 4. Zuo et al. showed that HMGB1 inhibits lung cancer cell metastasis by suppressing the activation of CREB and nWASP expression 6. Luan et al. reported that HMGB1 is negatively correlated with the development of endometrial carcinoma and prevents cancer cell invasion and metastasis by inhibiting epithelial-to-mesenchymal transition (EMT) 7. However, some studies have suggested that HMGB1 promotes cancer cell development and progression 8, 9. HMGB1/TLR4/myeloid differentiation factor 88 signalling promotes progression of GC 10. HMGB1 promotes GC cells proliferation and migration by activating the NF-кB and ERK signal pathways 11, 12. Although increasing evidence has shown that HMGB1 can induce the progression of GC 13, the underlying molecular mechanisms remain unclear.

We previously reported that HMGB1 expression levels were significantly higher in GC cells than in normal gastric epithelial cells, and knockdown of HMGB1 enhanced aloin-induced GC apoptosis 14. This finding indicated that HMGB1 was involved in GC progression. However, the exact mechanisms of HMGB1 in the proliferation and migration of GC remain elusive.

The present study investigated the effects of HMGB1 on GC proliferation and migration and explored the underlying molecular mechanisms involved. Our results showed that HMGB1 was expressed at higher levels in GC tissues and cells compared with adjacent non-tumour tissues and normal gastric epithelial cells, respectively. HMGB1 promoted GC HGC-27 cell proliferation and migration via RAGE-mediated Akt-mTOR and ERK-CREB signalling pathways. Furthermore, our data indicated that suppression of mTOR or ERK activation reduced the expression levels of RAGE, suggesting that the RAGE-mTOR/ERK feedback loop is involved in HMGB1-induced GC proliferation and migration. Taken together, our results highlight HMGB1 as a potential therapeutic target for GC and provide a novel perspective for the effect of HMGB1 on GC cell proliferation and migration.

## Materials and methods

### Antibodies and reagents

Rapamycin, LY294002, FPS-ZM1 and U0126 were purchased from Selleck Chemicals (Houston, TX, USA) and recombinant human HMGB1 (rhHMGB1) was provided by Sigma-Aldrich (Merck KGaA, Darmstadt, Germany). Human HMGB1 ELISA kits were purchased from CUSABIO (Wuhan, China), EdU proliferation detection kits were purchased from RiboBio Co., Ltd. (Guangzhou, China), primary antibodies against β-actin, p-Akt (Ser473), Akt, p-mTOR (Ser2448), mTOR, p-P70S6K (Thr421/Ser424), P70S6K, p-S6 (Ser240/244), S6, p-ERK (Thr202/Tyr204), ERK, p-P90RSK (Ser380), P90RSK-1, p-CREB (Ser133s), CREB, cyclin D1, cyclin E1, TLR2 and RAGE were purchased from Cell Signalling Technology (Beverly, MA, USA). HMGB1 antibody was purchased from AbCam (Cambridge, UK) and TLR4 antibody was provided by ABclonal Biotechnology Co., Ltd (Wuhan, China). E-cadherin, N-cadherin, MMP-9 and MMP-2 antibodies were obtained from Santa Cruz Biotechnology (Dallas, TX, USA). Secondary antibodies coupled to IRDye800 fluorophore for use with the Odyssey Infrared Imaging System were purchased from LI-COR Biosciences (Lincoln, Nebraska, USA). Horseradish peroxidase-conjugated anti-mouse IgG and anti-rabbit IgG secondary antibodies were obtained from Cell Signaling Technology (Beverly, MA, USA).

### Tissue microarray and immunohistochemistry (IHC) staining

GC adenocarcinoma and adjacent non-tumour tissue samples were obtained from 36 patients that underwent curative gastric cancer resection in the First Affiliated Yijishan Hospital of Wannna Medical College (Wuhu, Anhui, China). No patient received anti-tumor treatment before surgery. Sample preparation and experiment operation were conducted in accordance with ethical and legal standards, and the study was consented by the Yijishan Hospital of Wannan Medical College Ethics Committee. GC tissue microarray was produced by Service Biotechnology Co., Ltd. (Wuhan, China). HMGB1 levels were measured using HMGB1 antibody (diluted 1:200). A tissue chip scanner (Pannoramic MIDI, 3D HISTECH) was used to obtain tissue information. Briefly, Quant center is the analysis software for the Pannoramic viewer. After image scanning, the software densito Quant in Quant center was used to automatically identify and set all dark brown tissues to be strongly positive, brown yellow to be moderately positive, light yellow to be weakly positive and blue nuclei to be negative. The areas (in pixels) of strong positive, moderate positive, weak positive and negative, and the percentage of positive, were identified in each tissue point, and then histochemistry score (H-SCORE) was performed. H-SCORE = Σ(*Pi×I*) = (percentage of cells of weak intensity ×1) + (percentage of cells of intensive intensity ×2) + (percentage of cells of strong intensity×3), in which *Pi* indicates the percentage of positive cells in the total number of cells in the section; *I* represents the staining intensity. HMGB1 expression was semi-quantitatively estimated using the H-SCORE. The score ranges from 0 to 300, with higher scores representing a strong positive result. HMGB1 expression in GC tissue was categorised in advance into two groups: low expression HMGB1 (H-SCORE, <100) and high expression HMGB1 (H-SCORE, 100-300).

### Clinical parameters and data analysis

The clinic parameters of enrolled patients were shown in Table 1. The association of HMGB1 expression and the clinical parameters of GC patients were analysed using Fisher's exact test.

### Cell culture

HGC-27 cells and GES-1 normal gastric epithelial cells were purchased from GuangZhou Cellcook Biotech Co., Ltd. (GuangZhou, China). HGC-27 cells were maintained in RPMI-1640 medium (Gibco) containing 10% non-essential amino acids. GES-1 cells were cultured in DMEM medium (Gibco) supplemented with 10% foetal bovine serum (FBS; Lonsera, South America), 100 μg/mL streptomycin and 100 U/mL penicillin (Beyotime Institute of Biotechnology, Haimen, China). All cells were cultured in a humidified atmosphere with 5% CO_2_ at 37°C.

### Plasmids and transfection

HMGB1 overexpression (CMV‑MCS‑EGFP‑SV40‑Neomycin) and negative plasmids, HMGB1 interference (hU6-MCS-CMV-GFP-SV40-Neomycin) and negative plasmids were purchased from GeneChem Co., Ltd. (Shanghai, China). Cells were transfected with short hairpin RNA (shRNA) against HMGB1 with the following sequence: 5′-CGAAGAAACTGGGAGAGAT-3′. In brief, HGC-27 cells were seeded in 6-well plates until they reached 80% confluence. Next, transient transfection was performed using Lipofectamine 3000 Reagent (Thermo Fisher Scientific, Inc.) according to the manufacturer's instructions. The transfection efficiency was detected using Western blotting. Image J version 1.52 software (National Institutes of Health) was used for the densitometry analysis.

### Colony formation assay

After transfection, HGC-27 cells were seeded in 6-well plates and cultured in a humidified atmosphere with 5% CO_2_ at 37°C for 2 weeks. The culture supernatants were discarded and the cell colonies were fixed in the wells using 4% paraformaldehyde for 20 min and stained with 0.1% crystal violet for 30 min. After washing with phosphate-buffered saline (PBS), the cell colonies were observed and the numbers of colonies (≥25 cells/colony) were counted under a light microscope (Olympus, Japan).

### EdU assay

HGC-27 cells were seeded in 24-well plates and the cell proliferation ability was detected by EdU assay according to the manufacturer's instructions. Cells were observed using an inverted fluorescence microscopy (100× magnification; Olympus, Tokyo, Japan). The ratio of EdU-positive stained cells (red fluorescence) to DAPI-stained cells (blue fluorescence) was calculated.

### Wound healing assay

After treatment, HGC-27 cells were seeded in 12-well plates. When the cells reached monolayer confluence, the monolayer was scratched using a clean 200-μL pipette tip and the detached cells were removed by gently washing with PBS. Images were taken at 0 h and 24 h under a light microscopy (100× magnification; Olympus, Tokyo, Japan). The wound area was analysed using Image J version 1.52 software.

### Transwell assay

After treatment, HGC-27 cells were suspended in serum-free 1640 medium. A 200-μL cell suspension containing 2 × 10^4^ cells was seeded in the upper chamber of transwell places (Millipore, Billerica, MA, USA) and 600 μL of 1640 medium supplemented with 20% FBS was placed in the lower chamber. After culturing for 24 h at 37°C and 5% CO_2_, cells on the upper surfaces were gently removed using a cotton swab and cells that migrated to the lower surface were fixed using 4% paraformaldehyde for 20 min and stained with 0.1% crystal violet for 30 min at room temperature. Five randomly chosen fields were captured and photographed under an inverted microscope (100× magnification; Olympus, Japan). The number of migrated cells were counted using Image J version 1.52 software.

### Western blotting

Western blotting was performed according to the methods described previously by Wang et al. 15. Total protein was extracted from cells using radioimmunoprecipitation assay buffer (Beyotime Institute of Biotechnology, Haimen, China) supplemented with phenylmethylsulfonyl fluoride (PMSF). After 30 min lysing on ice, the amount of total protein was determined using a bicinchoninic acid (BCA) kit. Equal amounts of protein in each sample were separated using 12% SDS-PAGE and transferred to nitrocellulose membranes (Pall Corporation, Port Washington, USA). The membranes were then blocked with 5% skimmed milk at room temperature for 1 h, rinsed three times with TBST and incubated with the indicated primary antibodies overnight at 4°C. The membranes were then incubated with secondary antibody for 2 h at room temperature. Antibody-antigen complexes were visualised using either a LI-COR Odyssey Infrared Imaging System (LI-COR Biosciences) or a chemiluminescence imaging system (Clinx, Shanghai, China). Protein levels were semi-quantified using Image J 1.52 software.

### Statistical analysis

Experiments were performed in triplicate and data were shown as mean ± SD. The statistical significance of differences was performed using Student's *t* test for comparison of two groups or one-way analysis of variance (version 17.0 SPSS, Chicago, IL, USA) for comparison of more than two groups followed by Tukey's multiple comparison test. Fisher's exact test was used to analyse correlations between HMGB1 and clinicopathological factors of patients with GC. All *p*-values <0.05 were considered significant.

## Results

### HMGB1 was highly expressed in GC tissues and cells

**Figure 1:**
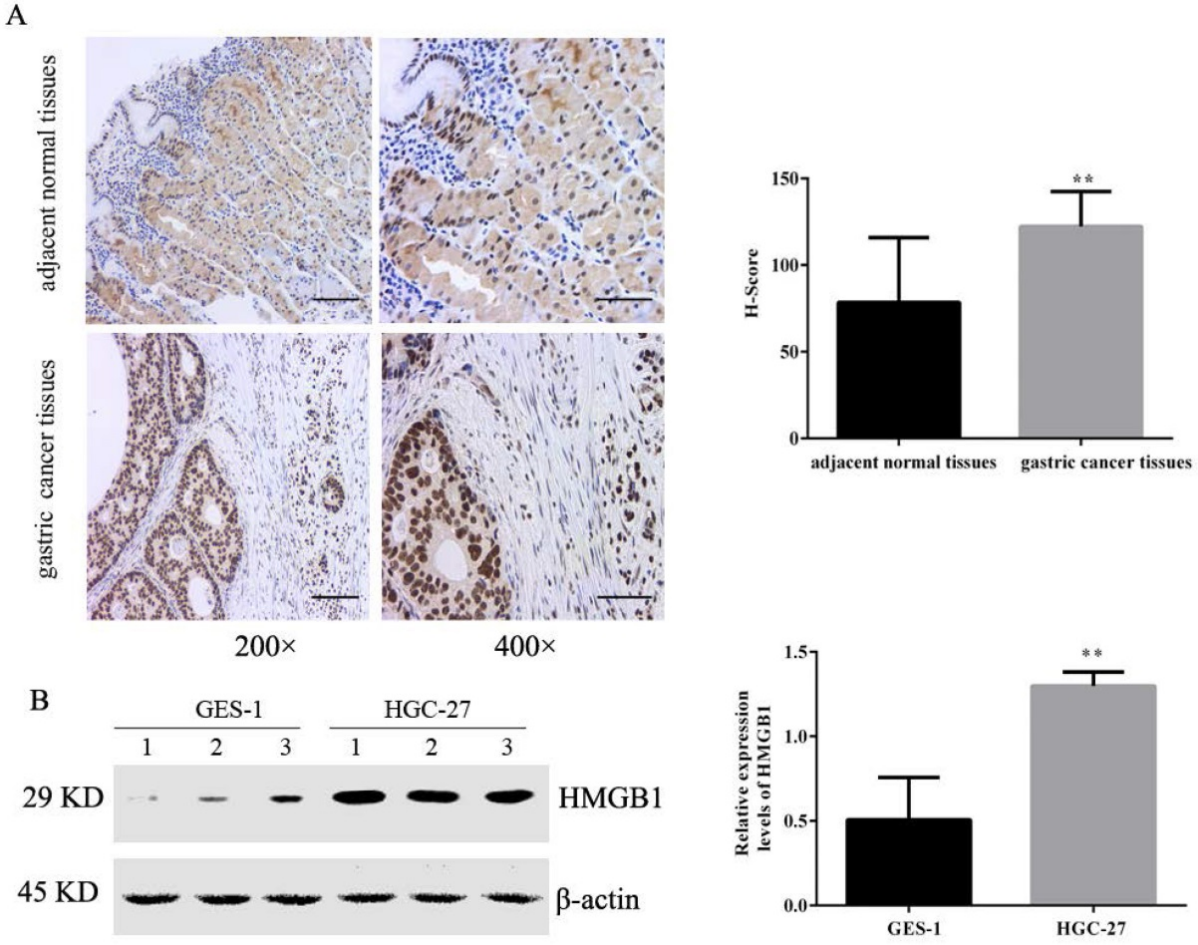
** HMGB1 was highly expressed in GC tissues and cells.** (**A**) Expression levels of HMGB1 in GC tissues and adjacent normal tissues were detected by IHC (left panel: magnification, 200×, scale bar: 50 µm; right panel: magnification, 400×, scale bar: 25 µm). (**B**) Expression levels of HMGB1 in GC cell lines (HGC-27) and normal gastric cell (GES-1) were determined using Western blotting. Data represent mean ± SD. * *p* < 0.05, ** *p* < 0.01 vs adjacent normal tissues or GES-1.

Tissue microarrays including 36 pairs of tumour and adjacent normal tissues were evaluated by IHC. The positive immunohistochemical staining of HMGB1 was dark brown, and the staining intensity and number of positive cells in cancer tissues was significantly higher than that of adjacent tissues. Semi-quantitative estimation using the H-SCORE showed that the positive expression of HMGB1 was higher in GC tissues than in the adjacent normal tissue (Fig. 1A). We also measured levels of HMGB1 in GC HGC-27 and normal gastric epithelial cells GES-1 using Western blotting. Expression of HMGB1 was significantly higher in GC cells compared with normal gastric epithelial cells (Fig. 1B), suggesting that HMGB1 may regulate GC progression.

### Correlation between HMGBl expression and Clinical parameters

GC patients were classified according to HMGBI immunoreactive intensity as low and high HMGB1 groups. The association between HMGB1 expression and the clinical parameters of the GC patients was analysed. As summarised in Table 1, our data indicated that there was no significant correlation between HMGB1 level and the clinicopathologic factors, such as age, gender, tumour size, TNM stage and histological grade (*p* > 0.05 for all).

### HMGB1 promotes colony formation and proliferation in GC cells

**Figure 2:**
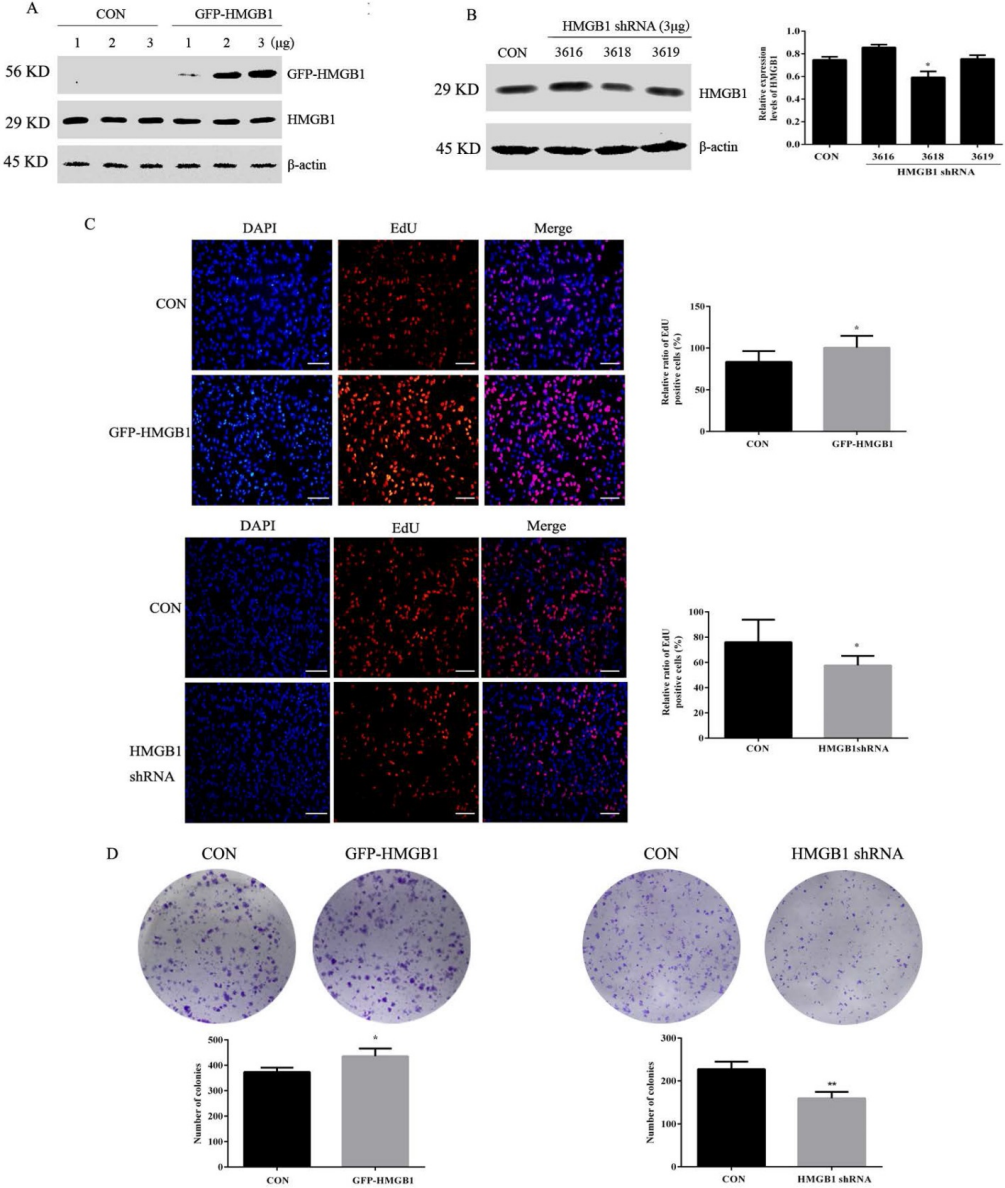
** HMGB1 promoted GC cell proliferation.** (**A**) HGC-27 cells were transfected with different doses of GFP-HMGB1 and negative plasmids for 24 h and HMGB1 expression was measured by Western blotting. (**B**) HGC-27 cells were transfected with HMGB1 shRNA and negative plasmids for 48 h and HMGB1 levels were measured by Western blotting. β-actin was used as a loading control. HGC-27 cells were transfected with 3 µg of GFP-HMGB1 for 24 h or HMGB1 shRNA plasmids for 48 h, with an equivalent negative plasmid as control respectively. EdU assay (magnification, 100×) (**C**) and colony formation assay (**D**) were used to measure cell proliferation. Experiments were repeated three times and data represent mean ± SD. *p < 0.05 and ** *p* < 0.01 vs cells transfected with negative plasmid.

To investigate the effects of HMGB1 on GC, HGC-27 cells were transfected with GFP-labelled HMGB1 overexpression plasmids (GFP-HMGB1) or HMGB1 shRNA interference plasmids (HMGB1 shRNA). The transfection efficiencies were verified using Western blotting. We selected 3 µg of HMGB1-GFP plasmid to transfect HGC-27 cells in subsequent experiments (Fig. 2A). Three shRNAs targeting HMGB1 (nos. 3616, 3618 and 3619) were constructed and transfected to HGC-27 cells. The interference efficiencies of the different plasmids are shown in Fig. 2B. HMGB1 shRNA no. 3618 exhibited obvious inhibitory effects; therefore, this plasmid was used for subsequent experiments.

Next, the effects of HMGB1 on GC proliferation were assessed using EdU and colony formation assays. EdU (5-ethyl-2'-deoxyuridine) is a thymine nucleoside analogue, which can replace thymine (T) and infiltrate into the replicating DNA molecules during the cell proliferation stage. EdU positive cells (red staining) represent proliferative cells, blue staining for total cells, the percentage of red staining cells in total cells represent GC proliferation ability. The data showed that overexpression of HMGB1 enhanced HGC-27 cell proliferation compared with cells transfected with the negative plasmids. Furthermore, downregulation of HMGB1 exhibited the opposite effect (Fig. 2C, D). These results suggested that HMGB1 enhanced the proliferation ability of HGC-27 cells.

### HMGB1 enhances GC cells migration

**Figure 3:**
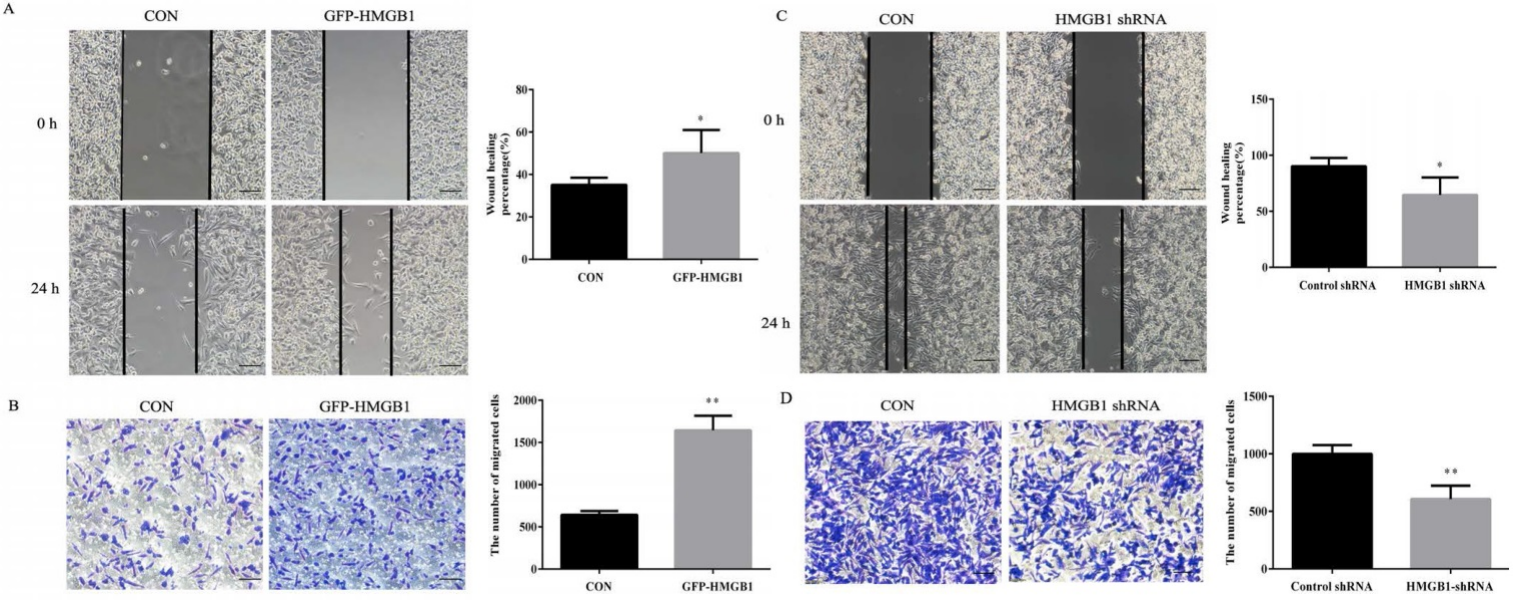
** HMGB1 enhanced GC cells migration.** HGC-27 cells were transfected with 3 µg of GFP-HMGB1 and negative plasmids for 24 h. Wound healing (**A**) and transwell (**B**) assays were used to determine cell migration. HGC-27 cells were transfected with 3 µg of HMGB1 shRNA and negative plasmids for 48 h and wound healing (**C**) and transwell (**D**) assays were used to detect cell migration, magnification, 100×. Each experiment was repeated three times and data represent mean ± SD. *p < 0.05 and ** *p* < 0.01 vs cells transfected with negative plasmid.

Wound healing and transwell assays were used to evaluate the effects of HMGB1 on GC migration. As shown in Fig. 3A, the healing rate of scratches clearly increased after GFP-HMGB1 transfection and the migration of HGC-27 cells was also increased following HMGB1 overexpression (Fig. 3B). However, HMGB1 knockdown noticeably decreased the wound healing rate and migration of cells (Fig. 3C, D). These findings indicated that HMGB1 increased HGC-27 cell migration.

### HMGB1 affects the expression levels of cyclins, MMPs and EMT markers

**Figure 4:**
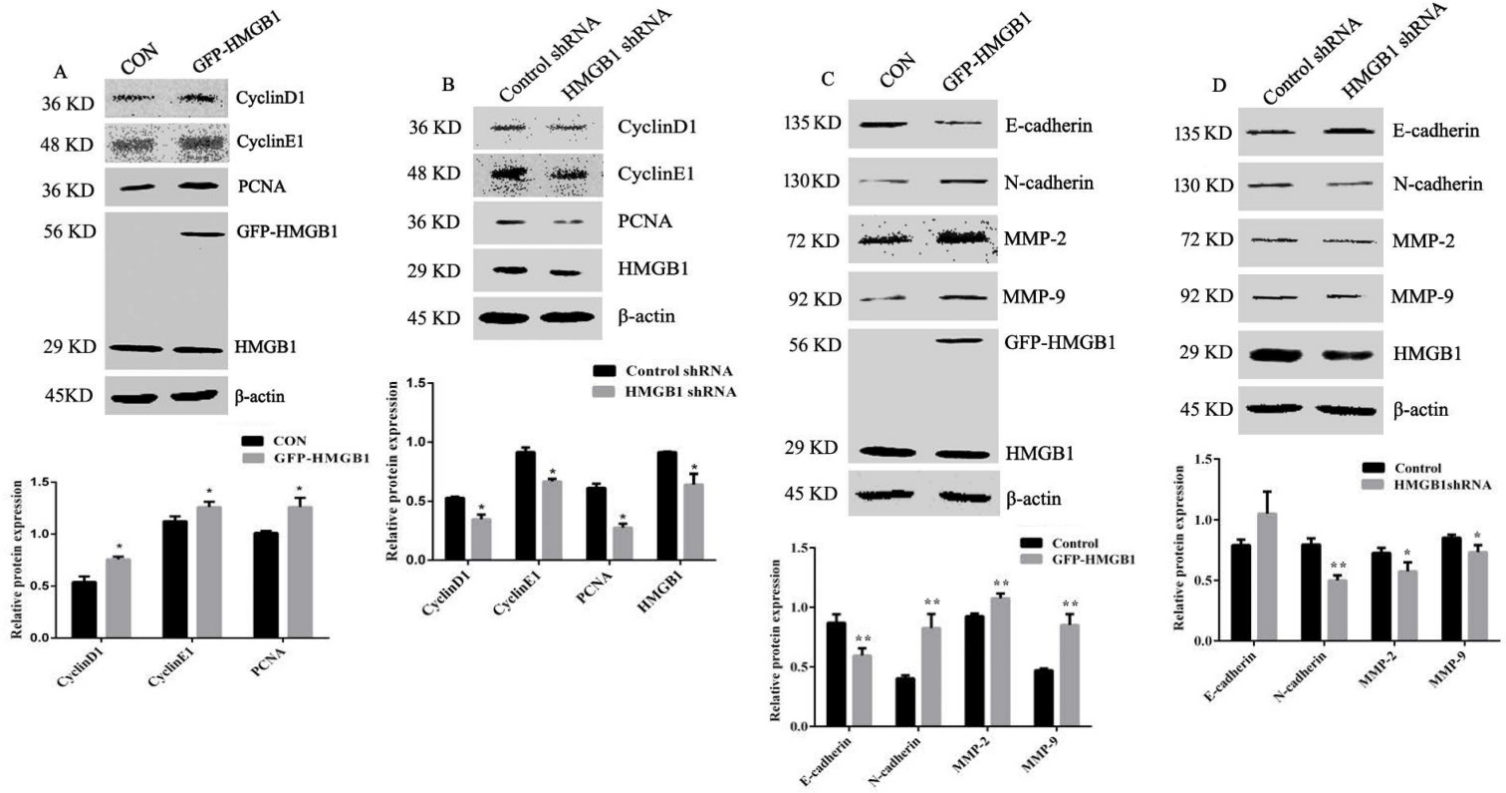
** HMGB1 upregulated the expression levels of cyclin D1, E1 and PCNA and downregulated expression of MMPs and EMT.** HGC-27 cells were transfected with GFP-HMGB1 or negative plasmids for 24 h (**A**) or HMGB1 shRNA and negative plasmids for 48 h (**B**) and levels of cyclin D1, E1 and PCNA were measured by Western blotting. HGC-27 cells were transfected with GFP-HMGB1 or negative plasmids for 24 h (**C**) or HMGB1 shRNA and negative plasmids for 48 h (**D**) and expression levels of N-cadherin, E-cadherin, MMP-2 and MMP-9 were determined by Western blotting. The relative levels were normalised to β-actin. Data represent mean ± SD of three independent experiments and the representative blots were shown. *p < 0.05 and **p < 0.01 vs cells transfected with negative plasmid.

To explore the mechanisms of HMGB1-mediated promotion of GC proliferation and migration, levels of cyclin D1, cyclin E1, PCNA, MMP-2, MMP-9, N-cadherin and E-cadherin were measured using Western blotting. The expression levels of cyclin D1, cyclin E1 and PCNA were increased following HMGB1 overexpression, but were attenuated following HMGB1 knockdown compared with cells transfected with negative plasmids (Fig. 4A, B). Measurement of MMP-2, MMP-9, N-cadherin and E-cadherin expression showed that overexpression of HMGB1 upregulated expression levels of N-cadherin and downregulated the expression levels of E-cadherin, MMP-2 and MMP-9, whereas knockdown of HMGB1 showed the opposite effect (Fig. 4C, D). Taken together, our data indicate that HMGB1 is able to promote the expression of cyclins and MMPs and enhance EMT in HGC-27 cells.

### HMGB1 promotes activation of Akt/mTOR/P70S6K and ERK/P90RSK/CREB signalling pathways

**Figure 5:**
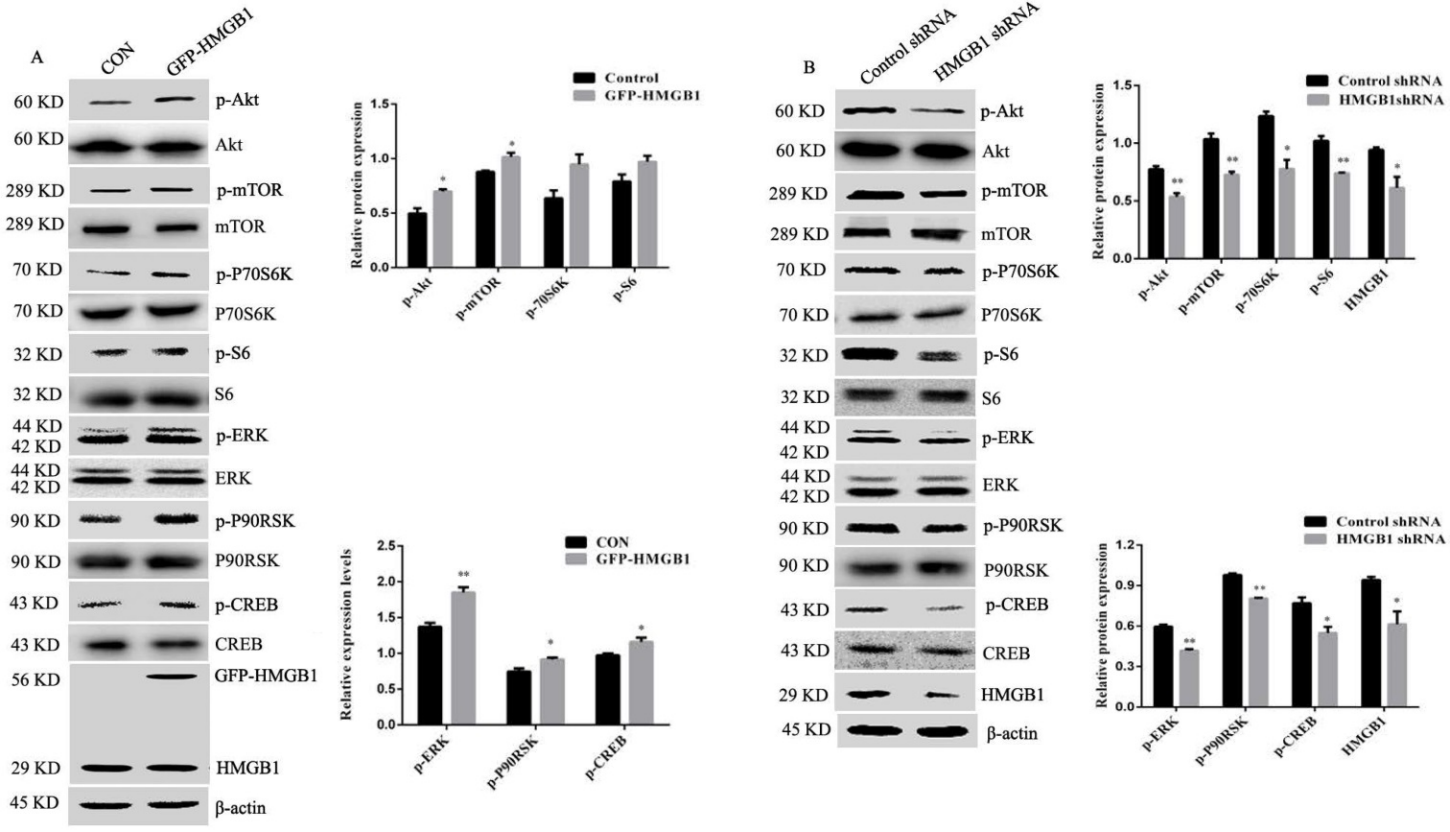
** HMGB1 upregulated phosphorylation of Akt and ERK signalling pathways.** HGC-27 cells were transfected with GFP-HMGB1 or negative plasmids for 24 h (**A**), or HMGB1 shRNA and negative plasmids for 48 h (**B**) and phosphorylation of Akt, mTOR, P70S6K, S6, ERK, P90RSK and CREB were measured by Western blotting. Their relative levels were normalised to total protein respectively. The relative levels of HMGB1 were normalised to β-actin. Data represent mean ± SD and the representative blots were shown. *p < 0.05 and **p < 0.01 vs cells transfected with negative plasmid.

Akt and ERK play an important role in cancer initiation and progression 16. To investigate the potential molecular mechanisms of HMGB1-mediated regulation of GC cell proliferation and migration, we measured the activation of Akt and ERK signalling pathways using Western blotting. Phosphorylation of Akt/mTOR and ERK/P90RSK/CREB were increased in HMGB1-overexpressed HGC-27 cells compared with cells transfected with the control plasmid, while the activation of these signalling pathways showed the opposite effects after transfection with HMGB1 shRNA. Phosphorylation of P70S6K and S6 were enhanced in response to HMGB1 overexpression, but there was no statistical difference compared with cells transfected with the control plasmid (Fig. 5A, B). Taken together, these data suggest that HMGB1 promotes GC cells proliferation and migration by enhancing the phosphorylation of Akt/mTOR and ERK signalling pathways in HGC-27 cells.

### HMGB1 upregulates the expression of RAGE but not TLR2 and TLR4

**Figure 6:**
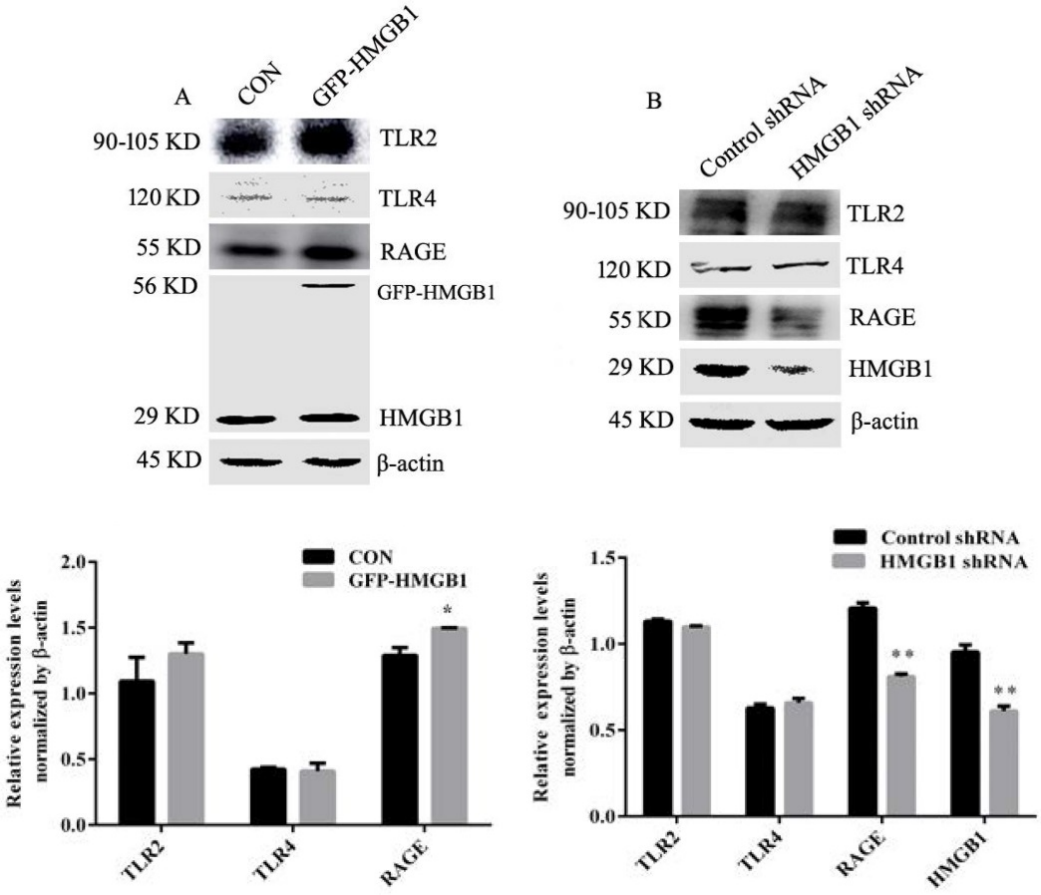
** HMGB1 promoted expression of RAGE but not TLR2 and TLR4.** HGC-27 cells were transfected with GFP-HMGB1 or negative plasmids for 24 h (**A**), or HMGB1 shRNA or negative plasmids for 48 h (**B**) and expression levels of RAGE, TLR1 and TLR4 were determined by Western blotting. Relative levels were normalised to β-actin. Data represent mean ± SD. *p < 0.05 and **p < 0.01 vs cells transfected with negative plasmid.

Extracellular HMGB1 participates in regulating a variety of biological behaviours by binding with receptors such as RAGE, TLR2 and TLR4 5, 10. We explored the effect of HMGB1 on its receptor expression. HGC-27 cells were transfected with HMGB1 overexpression or control plasmids and Western blotting was used to determine the levels of TLR2, TLR4 and RAGE. Overexpression of HMGB1 increased RAGE expression but not TLR2 and TLR4 (Fig. 6A), whereas knockdown of HMGB1 decreased the expression level of RAGE, but did not affect the expression of TLR2 and TLR4 (Fig. 6B). These results suggested that RAGE may mediate the activation of Akt and ERK signalling pathways induced by HMGB1.

### RAGE mediates HMGB1-induced Akt and ERK signalling pathway activation

**Figure 7:**
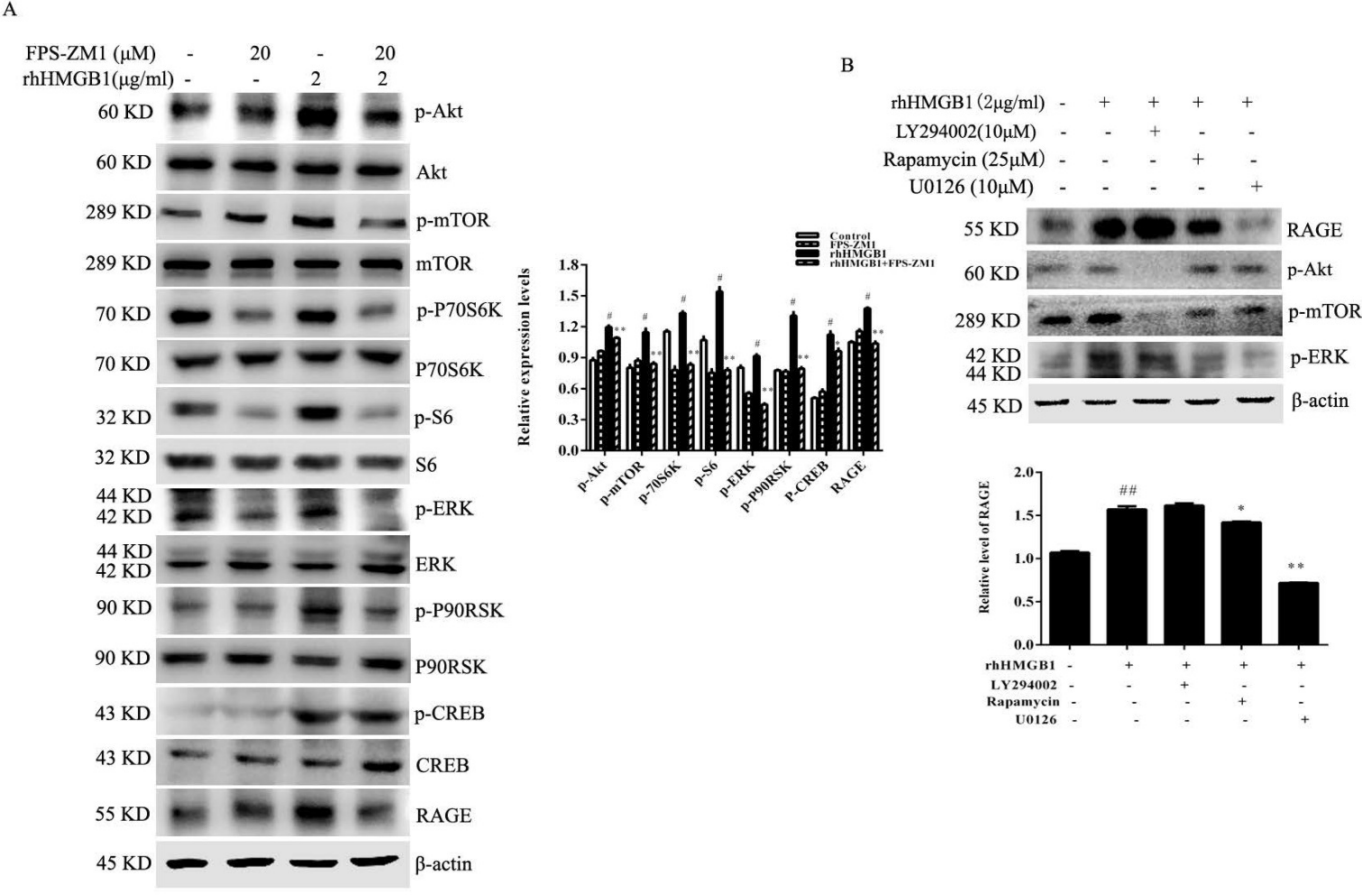
** RAGE-mediated rhHMGB1-induced activation of Akt/mTOR and ERK signalling pathways.** (**A**) HGC-27 cells were pretreated with the RAGE-specific inhibitor, FPS-ZM1, for 1 h and then stimulated with rhHMGB1 for 12 h. Phosphorylation of Akt, mTOR, P70S6K, S6, ERK and P90RSK were detected by Western blotting. Their relative levels were normalised to total protein respectively. (**B**) HGC-27 cells were pretreated with LY29402, rapamycin and U0126 for 1 h and then treated with rhHMGB1 for 12 h and RAGE expression and of Akt, mTOR and ERK were detected by Western blotting. The relative levels of RAGE were normalised to β-actin. Data represent mean ± SD. #*p* < 0.05 vs control group, *p < 0.05 and **p < 0.01 vs rhHMGB1-treated cells.

To determine whether RAGE receptor was responsible for HMGB1-induced Akt and ERK signalling pathway activation, HGC-27 cells were treated with rhHMGB1 (2 μg/mL) in the presence of the RAGE inhibitor, FPS-ZM1. rhHMGB1 enhanced the phosphorylation of Akt/mTOR/P70S6K and ERK/P90RSK signalling pathways and FPS-ZM1 pretreatment attenuated rhHMGB1-induced phosphorylation of these signalling pathways (Fig. 7A).

### Inhibition of mTOR and ERK activation attenuates rhHMGB1-induced RAGE expression

Although our results suggested that HMGB1 induced RAGE expression, the underlying molecular mechanism remained unclear. HGC-27 cells were pretreated with either LY294002, rapamycin or U0126 (specific inhibitors of Akt, mTOR and ERK, respectively) for 1 h prior to stimulation with rhHMGB1 for 12 h and measurement of RAGE level using Western blotting. Expectedly, our data showed that inhibition of mTOR and ERK activation significantly downregulated rhHMGB1-induced RAGE expression (Fig. 7B).

### Inhibition of Akt and ERK activation attenuates rhHMB1-induced HGC-27 cell proliferation and migration

**Figure 8:**
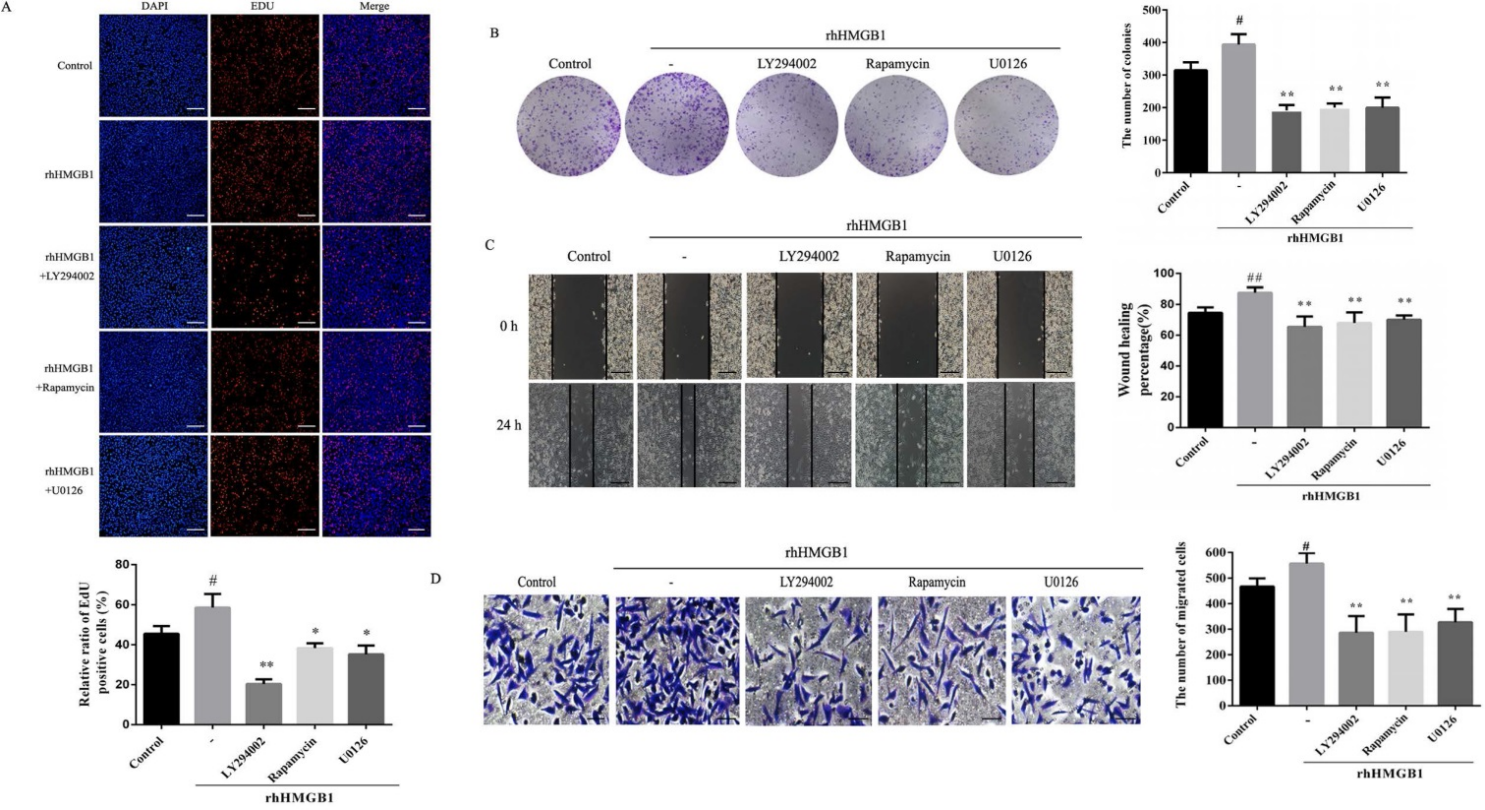
** Inhibition of Akt, mTOR and ERK activation suppressed rhHMGB1-induced proliferation and migration in HGC-27 cells.** HGC-27 cells were treated with LY29402, rapamycin and U0126 for 1 h and stimulated with rhHMGB1 for 12 h. EdU assay (**A**) and colony formation assay (**B**) were used to detect cell proliferation and wound healing (**C**) and transwell (**D**) assays were used to determine cell migration, magnification, 100×. All experiments were repeated three times and data represent mean ±SD. ^#^*p* < 0.05, ^##^*p* < 0.01 vs control group, *p < 0.05 and **p < 0.01 vs rhHMGB1-treated cells.

In order to further verify the role of Akt/mTOR and ERK in rhHMGB1-induced HGC-27 cell proliferation and migration, the effects of LY294002, rapamycin and U0126 on rhHMGB1-induced GC cell proliferation and migration were examined using EdU, colony formation, wound healing and transwell assays. Treatment with the inhibitors suppressed rhHMGB1-induced HGC-27 cell proliferation and migration (Fig. 8A-D). Taken together, the results suggested that HMGB1 regulated GC cell proliferation and migration via Akt/mTOR and ERK signalling pathways.

## Discussion

GC is one of the leading causes of cancer-related mortality worldwide 17. Its high aggressiveness and poor diagnosis are the main reasons for unsatisfactory therapeutic effects 18, 19. HMGB1 is reported to be highly expressed in many malignant tumours and is closely related to apoptosis, proliferation and migration of cancer cells 3, 13. Therefore, anticancer therapy targeting HMGB1 is attracting increased attention 20, 21.

In the present study, we measured the expression levels of HMGB1 in GC tissues and cells. As previously reported 22,23, we observed that HMGB1 was highly expressed in GC tissue compared with adjacent non-cancerous tissue (Fig. 1A). This suggested that HMGB1 was involved in the progression of GC. Therefore, we examined the association between HMGB1 and clinicopathological factors, but found no correlation (Table 1). This result was consistent with the findings reported previously by Zhang et al. 11. However, Suren et al. found that HMGB1 expression was significantly correlated with T stage and tumour differentiation 23. These differences may be due to differences in the evaluation of staining and the number of cases studied. HMGB1 expression levels in GC cells were also measure by Western blotting. Our results showed that HMGB1 expression was higher in GC cells compared with normal gastric epithelial cells (Fig. 1B).

Due to the high expression levels of HMGB1, HGC-27 cells were used in subsequent experiments. To determine the roles of HMGB1 in GC proliferation and migration, GFP-HMGB1 overexpression and HMGB1 shRNA plasmids were transfected in cells. Our results revealed that overexpression of HMGB1 promoted colony formation and proliferation ability in GC cell, whereas HMGB1 knockdown showed the opposite effects (Fig. 2). Both wound healing and transwell assays showed that the migration of HGC-27 cells was also enhanced by HMGB1 overexpression. Furthermore, downregulation of HMGB1 reduced the migration capacity of GC cells (Fig. 3). Our results are consistent with the findings from previous studies 11, 24.

PCNA plays a key role in the initiation and extension of replication and is an excellent inhibition target to shut down highly proliferative cells 25. Cyclins are essential proteins involved in cell cycle regulation and inhibition of cyclins expression reduces cell proliferation 26. To further examine the effects of HMGB1 on cell proliferation and migration, we determined the expression levels of cyclins and PCNA in HGC-27 cells. Overexpression of HMGB1 increased the expression levels of cyclin D1, E1 and PCNA, whereas HMGB1 knockdown reduced their expression (Fig. 4A, B). These data suggested that HMGB1 enhanced GC cell proliferation by regulation of cyclins expression and DNA replication. EMT is a key feature of tumour metastasis 27 and downregulation of MMP‑2 and MMP‑9 expression indicates cancer cell invasion and metastasis 28, 29. The expression levels of MMP-2, MMP-9 and EMT marker proteins were also measured in the present study. Our data showed that overexpression of HMGB1 enhanced MMP-2, MMP‑9 and N-cadherin levels and reduced E-cadherin expression, whereas knockdown of HMGB1 showed the opposite effects (Fig. 4C, D). Taken together, these results indicate that HMGB1 promotes GC cell migration by regulating EMT and MMP expression.

Extracellular HMGB1 mediates various responses by interacting with its cell surface receptors and triggering diverse biological effects, such as cell proliferation, migration, differentiation and apoptosis 30, 31. Akt/mTOR and ERK signalling pathways play an important role in cell proliferation, survival, growth and apoptosis 32-34. We examined the effects of HMGB1 on Akt/mTOR and ERK/CREB signalling pathway activation. Upregulation of HMGB1 enhanced phosphorylation of the Akt/mTOR and ERK signalling pathways. However, knockdown of HMGB1 caused a clear decrease in the activation of these signalling pathways (Fig. 5). HMGB1 overexpression enhanced the phosphorylation of P70S6K and S6, but was not significantly different. These may be due to high expression of HMGB1 in HGC-27 cells. Activation of P70S6K and S6 induced by endogenous HMGB1 is close to the maximum level.

To explore which receptor mediate the activation of Akt/mTOR and ERK signalling pathways induced by HMGB1, expression levels of RAGE, TLR2 and TLR4 were measured. Overexpression and knockdown of HMGB1 in HGC-27 cells revealed that HMGB1 upregulated expression levels of RAGE, but not TLR2 and TLR4 (Fig. 6). This suggested that RAGE may mediate HMGB1-induced Akt and ERK signalling pathways activation. To test this hypothesis, HGC-27 cells were pretreated with the RAGE inhibitor, FPS-ZM1, then stimulated using rhHMGB1 and measured phosphorylation of Akt and ERK signalling pathways using Western blotting. As expected, inhibition of RAGE suppressed rhHMGB1-induced Akt/mTOR and ERK signalling pathways activation (Fig. 7A). These data suggested that HMGB1 promoted HGC-27 cell proliferation and migration via RAGE-mediated Akt and ERK signalling pathways activation.

HMGB1 was reported to induce endothelial progenitor cell (EPC) migration via a RAGE-dependent PI3K/Akt signalling pathway; however, the ERK signalling pathway was not involved in HMGB1/RAGE-induced EPC migration 35. This difference in the signalling pathway may due to the different cell type studied. Consistent with our findings, HMGB1 was shown to promote prostate cancer development and metastasis by activating the Akt signalling pathway 35. To clarify the signalling pathway involved in HMGB1-induced RAGE expression, we used specific inhibitors of Akt, mTOR and ERK to block the activation of these signal molecules. Blocking mTOR or ERK activation clearly attenuated rhHMGB1-induced RAGE expression (Fig. 7B). This result indicated that rhHMGB1 induced RAGE expression via the mTOR and ERK signalling pathways. Thus, our data indicated that a RAGE-mTOR/ERK feedback loop was involved in HMGB-induced GC cell proliferation and migration.

To further demonstrate that the Akt/mTOR and ERK signalling pathways are involved in HMGB1-induced HGC-27 cell proliferation and migration, HGC-27 cells were treated with specific inhibitors of Akt, mTOR and ERK. Our result revealed that inhibition of Akt, mTOR and ERK reduced rhHMGB1-induced GC cells proliferation and migration (Fig. 8).

The biological effects of extracellular HMGB1 require its translocation from the nucleus to the cytoplasm; however, it is not known how HMGB1 transfers to the cytoplasm. It is unclear whether the tumour microenvironment affects the nuclear translocation of HMGB1 and whether nuclear translocation of HMGB1 is related to its post-translation modification. Future studies are required to elucidate the mechanisms.

**Figure 9:**
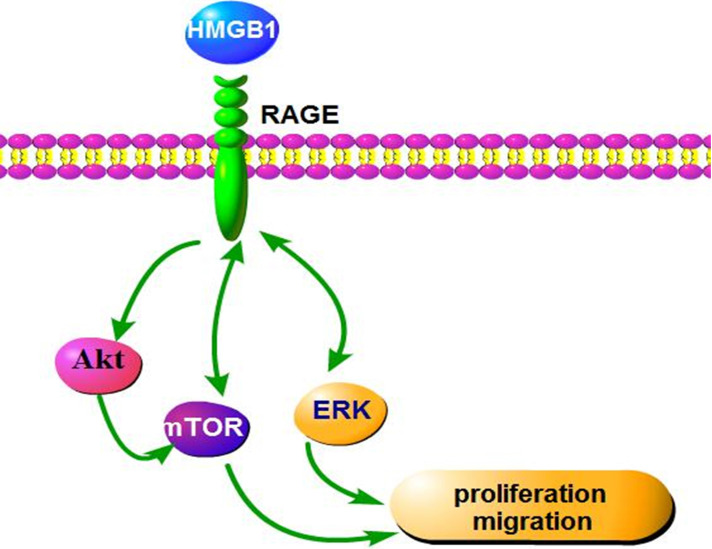
Schematic representation of the proposed mechanism of HMGB1 regulation of GC cell proliferation and migration.

In conclusion, our findings indicate that HMGB1 regulates GC cell proliferation and migration via activation of RAGE-mediated Akt/mTOR and ERK signalling pathways and a RAGE-mTOR/ERK feedback loop (Fig. 9). Our study provides a new perspective for the effect of HMGB1 on proliferation and migration of GC cells.

## Figures and Tables

**Table 1:** Correlation between HMGBl and clinical parameters. Fisher's exact test was used for statistical analysis

Parameter	Number	HMGB1 expression		*P* Value
		Low	High	
**Gender**				
Female	13	1	12	0.385
Male	23	5	18	
**Age (years)**				
≤65	24	4	20	1.000
>65	12	2	10	
**Tumor size (cm)**				
≤3	16	3	13	1.000
>3	20	3	17	
**Histological grade**				
Poorly differentiated	20	12	8	0.810
Moderately differentiated	6	3	3	
Highly differentiated	10	7	3	
**PTNM stage**				
Ta-T1	11	3	8	0.343
T2-T4	25	3	22	
